# Views of general practitioners on the role of CA125 in primary care to diagnose ovarian cancer

**DOI:** 10.1186/1472-6874-13-8

**Published:** 2013-02-19

**Authors:** Esther L Moss, Alison Moran, Timothy M Reynolds, Helen Stokes-Lampard

**Affiliations:** 1 University Hospitals of Leicester, Leicester, UK; 2 Mid Cheshire Hospitals NHS Foundation Trust, Crewe, UK; 3 Queen’s Hospital, Burton upon-Trent, UK; 4 Clinical Director PC-CRTU, Primary Care Clinical Sciences, University of Birmingham, Edgbaston, Birmingham, B15 2TT, UK

**Keywords:** Ovarian cancer, CA125, Tumour marker, Primary care

## Abstract

**Background:**

NICE guidance on the investigation and treatment of ovarian cancer advocates that the tumour marker CA125 should be the first line investigation for women suspected of having ovarian cancer.

**Methods:**

An internet-based survey, of primary care doctors in the West Midlands, was conducted in order to ascertain the views of general practitioners (GPs) of NICE guidance on the use of CA125 to triage suspected ovarian cancer cancers and the impact that this may have on referral pathways.

**Results:**

In total 258 GPs responded to the questionnaire. Although 219 (84.9%) responders reported awareness of the NICE guidance only 146 (56.6%) had personally read the document. The majority 187 (72.5%) of respondents anticipated that their use of CA125 would increase as a result of the new guidance. Abdominal bloating (>50 years), persistent abdominal distension and the presence of an abdominal or pelvic mass/swelling were the symptoms felt to be most associated with ovarian cancer. When questioned on the management of a woman with a raised CA125 the majority of respondents reported that a normal ultrasound scan would not stop an urgent secondary care referral if the CA125 was raised. There was no significant difference in the opinions of GPs with <5 years primary care experience compared to GPs with 6+ years.

**Conclusion:**

The symptoms associated with ovarian cancer are well understood by the GPs that responded however, a coordinated programme of education and training is needed for GPs on the role of CA125 in ovarian cancer, in addition to clearly defined referral pathways, in order to address a likely significant increase in suspected ovarian cancer referrals to secondary care, most of whom will not have ovarian cancer.

## Background

Recently published NICE guidance relating to the investigation and treatment of ovarian cancer [1] advocates increased use of serum CA125 in primary care, in order to improve the detection of ovarian cancer. Despite the large numbers needed to detect a single cancer, NICE concluded that, based on health economic evaluations, serum CA125 should be the first test performed on women with symptoms suggestive of ovarian cancer.

CA125 is an acute phase reactant which can be raised in benign processes as a result of inflammation or infection [2]. There are very few published studies investigating the role of CA125 in primary care as compared to secondary care and screening populations. CA125 has yet to be shown to function as an effective screening tool by detecting early stage disease or by reducing ovarian cancer mortality [3]. An understanding of the low sensitivity and false positive associations of CA125 amongst doctors working in surgical, medical and primary care specialties has previously been shown to be poor [4].

We conducted an internet-based survey of primary care doctors in the West Midlands, in October 2011. The aim of the study was to ascertain the views of general practitioners (GPs) of NICE guidance on the use of CA125 to triage suspected ovarian cancer cancers and the impact that this may have on referral pathways.

## Methods

A questionnaire was designed using the internet-based survey tool ‘Survey Monkey’ in order to facilitate completion and increase response rate whilst minimizing cost and the bias associated by imputing data from paper surveys (Additional file 1). Information was gathered on GPs’ personal clinical experience, the number of ovarian cancer cases they had managed over the past 2 years and their views on the symptoms associated with ovarian cancer. Two questions aimed to investigate GPs’ management preferences when presented with a woman reporting symptoms that may have been due to ovarian cancer.

The survey was piloted amongst a group of 10 General Practitioners with an interest in gynaecological malignancy, and their comments led to modifications of the survey prior to its general distribution. After discussion with the local research and development representative the study was classified as service evaluation and as such did not require ethical approval. A single email message containing a link to the survey was sent out in an email to all GPs on the Royal College of General Practitioners Midland Faculty database, by Faculty administrators to preserve GP confidentiality. No reminder emails were sent, no measurement of accuracy of the email database was available and no incentive for completion of the study was offered.

Results were collated automatically, analyzed and the responses of GPs with less than 5 years experience in primary care compared with GPs who had 6+ more years of experience by determining difference in confidence intervals for binomial proportions using the Normal approximation interval method.

## Results

In total 258 GPs responded to the questionnaire out of a total of 3,230 that were sent a single email explaining the project and containing a web link for the survey, giving a response rate of 8.0%.

The breakdown of the number of years in primary care and the location and demographic breakdown of the respondents’ practices is contained in Table 1. The number of ovarian cancer cases seen by the respondents over the previous 2 years was very low: 112 (43.4%) reported 2-4 cases, 70 (27.1%) one case and 60 (23.3%) no cases. The number of suspected cases referred to secondary care by the respondents over that same time period was also very low: 150 (58.1%) reported no referral, 70 (27.1%) one referral and 29 (11.2%) two referrals. CA125 was used to help with making the diagnosis in the majority of cases: 63 (42.6%) every time, 38 (25.7%) some of the time but not used 45 (30.4%) cases.

**Table 1:** Demographics of study population (n=258)

	**Number (percentage in brackets)**
**Gender**	
Male	99 (38.4)
Female	156 (60.5)
**Experience**	
GP in training	38 (14.7)
GP <5 years	72 (27.9)
GP 6-10 years	39 (15.1)
GP >10 years	109 (42.2)
**Location of practice**	
Rural	34 (13.2)
Suburban	66 (25.6)
Urban	102 (39.5)
Mixed	55 (21.3)
**Affluence of practice area**	
Affluent	46 (17.8)
Average	122 (47.3)
Deprived	88 (34.1)

Although 219 (84.9%) responders reported awareness of the NICE guidance only 146 (56.6%) had personally read the document. The majority, 187 (72.5%), anticipated that their use of CA125 would increase as a result of the new guidance.

The symptoms felt to be strongly associated with ovarian cancer were abdominal bloating (>50 years), persistent abdominal distension and the presence of an abdominal or pelvic mass/swelling (Table 2). These four symptoms also received the highest scores for the symptoms that would prompt the respondent to perform a CA125 (Table 3). When comparing the opinions of GPs with <5 years and with 6+ years experience in primary care there was no significant difference in their opinions on the symptoms associated with ovarian cancer or on the symptoms that would prompt a CA125.

**Table 2:** Responses to the question ‘What symptoms would make you consider ovarian cancer as a possible diagnosis?’

**Symptom**	**Yes definitely**	**Probably**	**Possibly**	**Very unlikely**	**No**
Abdominal pain	49 (21.5)	51 (23.3)	118 (51.8)	9 (3.9)	1 (0.4)
Bloating (<50 years)	35 (15.8)	38 (17.2)	110 (49.8)	36 (16.3)	2 (0.9)
Bloating (>50 years)	105 (46.1)	64 (28.1)	55 (24.1)	4 (1.8)	0
Abdominal distension (intermittent)	25 (11.2)	43 (19.2)	111 (49.6)	43 (19.2)	2 (0.9)
Abdominal distension (persistent)	144 (63.7)	51 (22.6)	31 (13.7)	0	0
Abdominal mass/swelling	148 (64.9)	52 (22.8)	25 (11.0)	3 (1.3)	0
Pelvic mass/swelling	194 (85.5)	25 (11.0)	7 (3.1)	1 (0.4)	0
Urinary frequency/urgency	26 (11.4)	52 (9.6)	119 (52.2)	29 (12.7)	2 (0.9)
Abnormal vaginal bleeding (pre/perimenopause)	31 (13.6)	47 (20.6)	96 (42.1)	50 (21.9)	4 (1.8)
Postmenopausal bleeding	38 (16.7)	33 (14.5)	97 (42.7)	51 (22.5)	8 (3.5)
Rectal bleeding	2 (0.9)	6 (2.7)	61 (27.0)	135 (59.7)	22 (9.7)
Loss of appetite	47 (20.7)	61 (26.9)	101 (44.5)	16 (7.0)	2 (0.9)
Unexplained weight loss	83 (36.4)	60 (26.3)	80 (35.1)	3 (1.3)	2 (0.9)

**Table 3:** Responses to the question ‘What symptoms would prompt you to request a CA125?’

**Symptom**	**Yes definitely**	**Probably**	**Possibly**	**Very unlikely**	**No**
Abdominal pain	33 (15.1)	45 (20.5)	106 (48.4)	22 (10.0)	13 (5.9)
Bloating (<50 years)	34 (15.7)	42 (19.4)	92 (42.6)	36 (16.7)	12 (5.6)
Bloating (>50 years)	105 (47.1)	69 (30.9)	39 (17.5)	4 (1.8)	6 (2.7)
Abdominal distension (intermittent)	23 (10.6)	53 (24.5)	98 (45.4)	31 (14.4)	11 (5.1)
Abdominal distension (persistent)	155 (69.5)	45 (20.2)	17 (7.6)	0	6 (2.7)
Abdominal mass/swelling	158 (70.9)	40 (17.9)	18 (8.1)	1 (0.4)	6 (2.7)
Urinary frequency/urgency	22 (9.9)	32 (14.3)	106 (47.5)	51 (22.9)	12 (5.4)
Abnormal vaginal bleeding (pre/perimenopause)	20 (9.1)	36 (16.4)	92 (42.0)	58 (26.5)	13 (5.9)
Postmenopausal bleeding	28 (12.7)	35 (15.8)	79 (35.7)	62 (28.1)	17 (7.7)
Rectal bleeding	1 (0.5)	4 (1.8)	52 (23.6)	124 (56.4)	39 (17.7)
Loss of appetite	44 (20.0)	46 (20.9)	101 (45.9)	20 (9.1)	9 (4.1)
Unexplained weight loss	75 (34.2)	68 (31.1)	64 (29.2)	6 (2.7)	6 (2.7)

The highest rated additional factors which respondents felt would make ovarian cancer more likely were: being postmenopausal, a family history of breast or ovarian cancer or a personal history of breast cancer (Table 4). GPs with 6+ years of experience did not rate breast cancer as great an associated factor as did GPs with less experience: scores for ‘yes definitely’ for personal history of breast cancer 39% (95% CI 30.9-47.9%) versus 63% (95% CI 52.8-72.2%) (95% CI for difference in proportion 6.2-40.1%) and for family history of breast cancer 27% (95% CI 19.2-34.7%) versus 53% (95% CI 42.5-62.8%) (95% CI for difference in proportion 8.9-42.5%) for 6+ years compared to <5 years respectively.

**Table 4:** General practitioners’ views on ‘What additional factors would make you consider ovarian cancer to be more likely?’

	**Yes definitely**	**Probably**	**Possibly**	**Very unlikely**	**No**
Postmenopausal	111(50.0)	71 (32.0)	28 (12.6)	8 (3.6)	4(1.8)
Early menopause	20 (9.4)	34 (16.0)	77 (36.3)	54 (25.5)	27 (12.7)
History of endometriosis	6 (2.8)	11 (5.1)	77 (35.5)	86 (39.6)	37 (17.1)
History of infertility	23 (10.6)	30 (13.8)	81 (37.3)	56 (25.8)	27 (12.4)
Nulliparity	48 (22.3)	42 (19.5)	88 (40.9)	25 (11.6)	12 (5.6)
Multiparity	1 (0.5)	7 (3.3)	58 (27.0)	105 (48.8)	44 (20.5)
FH ovarian cancer	171 (75.7)	37 (16.4)	18 (8.0)	0	0
PH breast cancer	110 (49.3)	66 (29.6)	42 (18.8)	3 (1.3)	2 (0.9)
FH breast cancer	83 (37.9)	66 (30.1)	58 (26.5)	10 (4.6)	2 (0.9)
Obesity (BMI>40)	57 (25.8)	80 (36.2)	66 (29.9)	13 (5.9)	5 (2.3)
HRT use	17 (7.7)	31 (14.1)	86 (39.1)	59 (26.8)	27 (12.3)
Previous hormonal contraception	5 (2.3)	19 (8.6)	48 (21.7)	97 (43.9)	52 (23.5)
No previous hormonal contraception	18 (8.2)	25 (11.4)	84 (38.2)	56 (25.5)	37 (16.8)

FH=family history, PH=personal history, HRT = hormone replacement therapy.

The majority of respondents reported that they would perform a speculum/pelvic examination of a patient in whom ovarian cancer was suspected prior to referral, 88 (38.3%) every time, 96 (41.7%) most cases, 39 (17.0%) rarely compared to 7 (3.0%) never. There was no significant difference in the answers given between the less and more experienced GPs, χ^2^ = 1.75; p=0.94.

When investigating the management of a woman presenting with persistent abdominal bloating for the past 1 month, who has no family history of ovarian cancer, the majority of GPs reported that they would initiate investigation in the community for a mildly raised CA125 (50) but that a more abnormal CA125 (200) would result in a referral to secondary care without out waiting for an ultrasound scan of the pelvis (Table 5). Even if an ultrasound scan of the pelvis demonstrated no abnormality, only a small proportion of GPs would be happy to watch and wait if the CA125 was raised and, if the CA125 was 200, over half would refer women using the suspected cancer pathway.

**Table 5:** General practitioners’ views on the management of a woman with no family history of ovarian cancer presenting with persistent abdominal bloating for the past 1 month

	**General gynaecology referral**	**Two week wait/ gynaecological oncology referral**	**Order USS**	**Watch and wait**
Premenopausal (<50 years)				
CA125 - 50	13 (6.7)	36 (18.5)	140 (71.8)	4 (2.1)
CA125 - 200	13 (6.7)	126 (64.9)	53 (27.3)	0
CA125 - 50/USS nad	63 (33.0)	41 (21.5)	-	36 (18.8)
CA125 – 200/USS nad	49 (25.5)	113 (58.9)	-	2 (1.0)
CA125 nad/USS nad	19 (9.7)	8 (4.1)	-	51 (25.6)
Postmenopausal (>50 years)				
CA125 - 50	19 (10.3)	53 (28.8)	107 (58.2)	4 (1.6)
CA125 - 200	10 (5.4)	133 (71.9)	41 (22.2)	0
CA125 - 50/USS nad	71 (38.8)	51 (27.9)	-	23 (12.6)
CA125 – 200/USS nad	32 (17.6)	112 (61.5)	-	2 (1.1)
CA125 nad/USS nad	23 (12.8)	13 (7.2)	-	28 (15.6)

USS = ultrasound scan, nad = no abnormality detected, 2 week wait = suspected cancer referral to gynaecology/gynaecolgical oncology.

## Discussion

Ovarian cancer was previously known as the ‘silent killer’ but studies have shown that it is associated with symptoms in 93% of cases before diagnosis [5]. The positive predictive value of individual symptoms, however, is very low, including the symptoms highlighted in this study as being ‘yes definitely’ associated with ovarian cancer, abdominal bloating 0.01-0.3% [6-9], abdominal distension 0.07-2.26% [6-10] and abdominal mass/swelling 0.48-11% [9,10]. Algorithms have been introduced to try and improve detection using symptoms. The Goff symptom index [11] has been shown to increase the specificity for the detection of ovarian cancer as compared to any symptoms but this is at the expense of sensitivity. In this study the GPs that responded to this questionnaire were aware of the typical symptoms associated with ovarian cancer (bloating (>50 years), persistent abdominal distension and abdominal/pelvic mass or swelling) and it is these symptoms that would prompt them to order a CA125. The symptom of ‘altered bowel habit’ was not specifically included in the questionnaire since it was not possible to include an exhaustive list of all the symptoms associated with ovarian cancer and the GP pilot group felt that this was more likely to prompt a referral to investigate the possibility of a colorectal malignancy.

The aim of questioning GPs on their management of a woman with no family history of ovarian cancer presenting with persistent abdominal bloating for the past 1 month was to try and determine their views and practice on referral pathways at different levels of CA125. We have shown that the higher the CA125 and being postmenopausal would increase the likelihood of referral to secondary care. We have also shown that although they would be happy to order an ultrasound to investigate a mildly raised CA125 (50), a higher result (200) would prompt a direct referral on a suspected cancer pathway without waiting for imaging. Our results show that a normal ultrasound would not stop referrals to secondary care if CA125 was raised, the majority stating that they would use a suspected cancer pathway if the CA125 was 200. This is in contrast to the NICE ‘Detection in primary care’ algorithm [12], which advises that a CA125 ≥35lU/ml and an ultrasound of the abdomen and pelvis suggestive of ovarian cancer should result in an urgent referral. The advised pathway of assessment for other clinical causes of a raised CA125 does not appear to be the management option of choice and may reflect the fact that CA125 is primarily known as a tumour marker for ovarian cancer and its false positive associations are not well known by GPs [4].

The recently introduced guidance has given cause for concerns from both the oncology and primary care communities. Gynaecological oncologists highlighted the poor sensitivity of CA125 in early stage disease and in non-serous subtypes [13], which could result in false reassurance from a normal value and in turn would delay rather than expedite a diagnosis [14]. Cave [15] reported on behalf of general practitioners by asking for guidance to help in the interpretation of CA125 in primary care. He also highlighted the lack of general practitioner involvement in development of the guidance, only one representative on the NICE guideline development group compared to six secondary care specialists. Although the reasoning behind the new guidance was logical, in an attempt to reduce the time from first presentation to diagnosis of ovarian cancer, there remain serious concerns. Without a fuller understanding of the true potential of CA125 as a tumour marker in primary care and support for general practitioners on its interpretation, the number of women referred to secondary care with suspected ovarian cancer will increase dramatically, as has been highlighted by this study. This will either require greater resources in secondary care to investigate or potentially undermine the rapid access service for “real cases”. There is also the concern that a normal CA125 result may now be taken as having excluded ovarian cancer and therefore further investigations or imaging will be delayed or not be undertaken until the disease is further advanced. On the other hand, it is becoming increasingly accepted that the most common and lethal subtype of ovarian cancer, high-grade serous, is likely to originate from the fallopian tube rather than the ovary. This would have a dramatic effect on the rationale for screening since detecting an abnormality in the ovary would be identifying metastatic disease and as such would not alter the long-term outcome if the diagnosis was delayed while waiting for investigations.

It is important to be mindful of the patient in the midst of this process. Calculations by NICE estimate that around 1 in 100 women with a positive CA125 or ultrasound scan will have ovarian cancer [12], therefore for every 100 women referred, 99 will not have ovarian cancer. The negative psychological impact on women referred needs to be considered since many will be informed that a raised CA125 means that they probably have ovarian cancer.

This study was a pragmatic, single email invitation on-line survey and, as such, has a very low response rate. The combination of no financial incentive, a rare clinical presentation in Primary Care and no follow up or reminder emails all contributed to the low response rate. Therefore this study cannot be taken as being representative of the views of GPs as a whole, however, it is reasonable to assume that the majority of GPs taking the time to complete the survey had a greater than average interest in the subject matter and consequently probably represent a ‘better informed’ GP population overall. Thus our findings are likely to be conservative and represent best current practice. Surveys are always subject to bias and we have not used any validated questionnaires or instruments, however we believe that we have revealed important and useful information about current thinking in primary care. This has a direct impact on increasing secondary care referrals and thus should inform future research into this subject.

## Conclusion

In conclusion, the use of CA125 as a primary diagnostic tool for suspected ovarian cancer has yet to be shown to be of benefit in a clinical trial, either in the primary or secondary care setting. The introduction of NICE guidance advocating its use is both premature and associated with potential risks. Without a coordinated programme of education and training for GPs, in addition to clearly defined referral pathways, it is likely that there will be a significant increase in suspected ovarian cancer referrals to secondary care, most of whom will not have ovarian cancer. Without the benefit of trial data only time will tell whether such a policy will actually improve the early detection and therefore survival of ovarian cancer patients or merely result in increased investigation and anxiety in many women.

## Competing interest

The authors declare that they have no competing interests.

## Authors’ contribution

EM conceived the idea for the study. EM, HS-L and TR designed the questionnaire and conducted the study. EM and AM analysed the results. TR performed the statistical analysis. EM drafted to manuscript. All authors contributed to revising the manuscript and approved its final contents.

## Pre-publication history

The pre-publication history for this paper can be accessed here:

http://www.biomedcentral.com/1472-6874/13/8/prepub

## Supplementary Material

Additional file 1 Questionnaire. Click here for file
