# Supplementary material for: Views of general practitioners on the role of CA125 in primary care to diagnose ovarian cancer
Source: BMC Womens Health. 2013 Feb 19;13:8. doi: 10.1186/1472-6874-13-8 (PMC3644283; doi:10.1186/1472-6874-13-8)
Supplement: Additional file 1 — Questionnaire. [file 1472-6874-13-8-S1.pdf]

Please tick the box that applies most closely for each question:

1. What stage of training are you at?

- Medical student
- Foundation Year 1/2
- GP in training
- Qualified GP 0-5 years
- Qualified GP 6-10 years
- Qualified GP >10 years

2. How would you describe the practice area in which you spend most of your clinical time?

- Rural
- Suburban
- Urban
- Mixed

3. How would you describe the deprivation or affluence in the area in which you spend most of your clinical time?

- Affluent
- Average
- Deprived

4. Are you male or female?

- Male
- Female

5. Over the past 2 years how many patients with ovarian cancer have you seen in primary care?

- 0
- 1
- 2-4
- 5-10
- >10

6. Over the past 2 years how many cases of primary ovarian cancer have you personally diagnosed or made the initial referral to secondary care?

- ◯ 0
- ◯ 1
- ◯ 2
- ◯ 3
- ◯ 4
- ◯ >4

7. Did you perform a CA125 to help with the diagnosis?

- ◯ Yes every time
- ◯ Yes some of the time
- ◯ No
- ◯ Don't know
- ◯ Not applicable

8. Are you aware of the NICE guidance on Ovarian Cancer published in April 2011?

- ◯ Yes
- ◯ No

9. Have you personally read the NICE guidance on Ovarian Cancer published in April 2011?

- ◯ Yes
- ◯ No

10. Do you anticipate that your CA125 use will increase following the publication of the NICE guidelines?

- ◯ Yes
- ◯ No
- ◯ Don't know

The following are a series of questions asking for your views of the role of CA125 in primary care.

For the following questions please tick all that apply:

11. What symptoms would make you consider ovarian cancer as a possible diagnosis?

	Yes definitely	Probably	Possibly	Very unlikely	No
Abdominal pain	◯	◯	◯	◯	◯
Bloating (<50 years)	◯	◯	◯	◯	◯
Bloating (>50years)	◯	◯	◯	◯	◯
Abdominal distension (intermittent)	◯	◯	◯	◯	◯
Abdominal distension (persistent)	◯	◯	◯	◯	◯
Adominal mass/swelling	◯	◯	◯	◯	◯
Pelvic mass/swelling	◯	◯	◯	◯	◯
Urinary frequency/urgency	◯	◯	◯	◯	◯
Abnormal vaginal bleeding (pre/perimenopause)	◯	◯	◯	◯	◯
Postmenopausal bleeding	◯	◯	◯	◯	◯
Rectal bleeding	◯	◯	◯	◯	◯
Loss of appetite	◯	◯	◯	◯	◯
Unexplained weight loss	◯	◯	◯	◯	◯

12. Are there any other symptoms you particularly associate with ovarian cancer?

13. What additional factors would make you consider ovarian cancer to be more likely?

	Yes definitely	Probably	Possibly	Very unlikely	No
Postmenopausal	◯	◯	◯	◯	◯
History of early menopause	◯	◯	◯	◯	◯
History of endometriosis	◯	◯	◯	◯	◯
History of infertility	◯	◯	◯	◯	◯
Nulliparity	◯	◯	◯	◯	◯
Multiparity	◯	◯	◯	◯	◯
Family history of ovarian cancer	◯	◯	◯	◯	◯
Personal history of breast cancer	◯	◯	◯	◯	◯
Family history of breast cancer	◯	◯	◯	◯	◯
Obesity (BMI>40)	◯	◯	◯	◯	◯
Hormone replacement therapy use	◯	◯	◯	◯	◯
Previous hormonal contraception use	◯	◯	◯	◯	◯
No previous hormonal contraception use	◯	◯	◯	◯	◯

14. What symptoms would prompt you to request a CA125?

	Yes definitely	Probably	Possibly	Very unlikely	No
Abdominal pain	◯	◯	◯	◯	◯
Bloating (<50 years)	◯	◯	◯	◯	◯
Bloating (>50 years)	◯	◯	◯	◯	◯
Abdominal distension (intermittent)	◯	◯	◯	◯	◯
Abdominal distension (persistent)	◯	◯	◯	◯	◯
Abdominal mass/swelling	◯	◯	◯	◯	◯
Urinary frequency/urgency	◯	◯	◯	◯	◯
Abnormal vaginal bleeding (pre/perimenopause)	◯	◯	◯	◯	◯
Postmenopausal bleeding	◯	◯	◯	◯	◯
Rectal bleeding	◯	◯	◯	◯	◯
Loss of appetite	◯	◯	◯	◯	◯
Unexplained weight loss	◯	◯	◯	◯	◯

15. If you suspected ovarian cancer in a woman >50 years would you order any tumour markers in addition to CA125?

	Yes	No	Don't know	Never heard of it
CEA	○	○	○	○
CA19.9	○	○	○	○
CA15.3	○	○	○	○
HCG	○	○	○	○
AFP	○	○	○	○
Inhibin	○	○	○	○
HE4	○	○	○	○

16. If the woman was under the age of 40 years would you order any tumour markers in addition to CA125?

	Yes	No	Don't know	Never heard of it
CEA	○	○	○	○
CA19.9	○	○	○	○
CA15.3	○	○	○	○
HCG	○	○	○	○
AFP	○	○	○	○
Inhibin	○	○	○	○
HE4	○	○	○	○

17. Would you perform a speculum/vaginal examination on a patient you were referring to secondary care for suspected ovarian cancer?

- ☐ Every case
- ☐ Most cases
- ☐ Rarely
- ☐ Never

18. If a patient had a raised CA125 and an ovarian cyst on ultrasound scan would you inform them that they had a high risk of having ovarian cancer? (assuming the normal range locally is <35iunits).

	Yes definitely	Yes	Maybe	Very unlikely	No
If CA125 was <35	○	○	○	○	○
If CA125 was 35-50	○	○	○	○	○
If CA125 was 51-100	○	○	○	○	○
If CA125 was 101-500	○	○	○	○	○
If CA125 was 501-1000	○	○	○	○	○
If CA125 was >1000	○	○	○	○	○

For the following case scenarios please select the letter corresponding to the management option that you think most closely fits what you would chose to do first:

a) Refer to gynae oncology directly

b) Refer to general gynaecology

c) Refer under 2 week wait guidelines to gynaecology

d) Refer to another hospital specialist

e) Do other blood tests

f) Order a pelvic ultrasound

g) Watch and wait

19. A premenopausal woman (<50 years) with no family history of ovarian cancer presents with persistent abdominal bloating for the past 1 month. What would you do:

	a	b	c	d	e	f	g
If her CA125 was 50	◯	◯	◯	◯	◯	◯	◯
If her CA125 was 200	◯	◯	◯	◯	◯	◯	◯
If her CA125 was 50 but a pelvic ultrasound scan showed no abnormality	◯	◯	◯	◯	◯	◯	◯
If her CA125 was 200 but a pelvic ultrasound scan showed no abnormality	◯	◯	◯	◯	◯	◯	◯
If her CA125 was normal and a pelvic ultrasound showed no abnormality but the abdominal bloating persisted	◯	◯	◯	◯	◯	◯	◯

20. If you would take a different course of management outside the options given above please comment in the box below.

21. A postmenopausal woman (>50 years) with no family history of ovarian cancer presents with persistent abdominal bloating for the past 1 month. What would you do?

	a	b	c	d	e	f	g
If her CA125 was 50	○	○	○	○	○	○	○
If her CA125 was 200	○	○	○	○	○	○	○
If her CA125 was 50 but a pelvic ultrasound scan showed no abnormality	○	○	○	○	○	○	○
If her CA125 was 200 but a pelvic ultrasound scan showed no abnormality	○	○	○	○	○	○	○
If her CA125 was normal and a pelvic ultrasound showed no abnormality but the abdominal bloating persisted	○	○	○	○	○	○	○

22. If you would take a different course of management outside the options given above please comment in the box below.

23. Did you refer to the NICE guidance when completing this questionnaire?

○ Yes

○ No

24. Thank you for taking the time to respond to this questionnaire which will be use as the basis of further research into ovarian cancer screening in primary care.

If you have any comments on the role of CA125 in primary care please use the box below.
